# Evaluation and Comparison of Crestal Bone Loss Around Dental Implants Placed Using Conventional Drilling, Bone Expansion, and Ridge Split Techniques: An In Vivo Study

**DOI:** 10.7759/cureus.106119

**Published:** 2026-03-30

**Authors:** Keshav Goyal, Sukant Sahoo, Aakarshan Dayal Gupta, Supriya Dahiya, Prerika Agarwal, Shivam Katyal, Rahul Verma, Sanjoy Dutta, Riya Phade, Seema Gupta

**Affiliations:** 1 Department of Prosthodontics and Crown and Bridge, Shree Bankey Bihari Dental College and Research Centre, Ghaziabad, IND; 2 Department of Prosthodontics and Crown and Bridge, Kothiwal Dental College and Research Centre, Moradabad, IND; 3 Department of Prosthodontics, Dr. Verma’s Dental Clinic and Implant Centre, New Delhi, IND; 4 Department of Prosthodontics, Giri Multi-Speciality Clinic, Hojai, IND; 5 Department of Orthodontics, Kothiwal Dental College and Research Centre, Moradabad, IND

**Keywords:** alveolar bone loss, bone expansion, cone-beam computed tomography, conventional, dental implants, ridge split

## Abstract

Introduction: Tooth loss is often followed by alveolar ridge resorption, which may compromise implant placement and long-term stability. Different implant site preparation techniques, such as conventional drilling, bone expansion, and ridge split procedures, are used to manage varying ridge conditions. These techniques may influence peri-implant bone remodeling in different ways. This study aimed to evaluate and compare crestal bone loss around dental implants placed using conventional drilling, bone expansion, and ridge split techniques.

Materials and methods: This prospective clinical comparative study included 72 implant sites in patients aged 25-55 years who required implant-supported rehabilitation. The implant sites were divided into three groups based on the implant site preparation technique used: conventional drilling (CD), bone expansion (BE), and ridge split (RS), with 24 implants in each group. Crestal bone levels were evaluated using cone-beam computed tomography immediately after implant placement and at 3, 6, and 12 months of follow-up. Mesial and distal crestal bone loss were measured and analyzed statistically using one-way analysis of variance (ANOVA) with Tukey’s post hoc test for intergroup comparisons and repeated-measures ANOVA for intragroup comparisons. Categorical variables were compared with the chi-square test. Statistical significance was set at p < 0.05.

Results: Crestal bone loss increased progressively over time in all three groups. Intergroup comparison revealed statistically significant differences in both mesial and distal crestal bone loss at 3, 6, and 12 months (p < 0.001). The ridge split group demonstrated the highest bone loss, followed by the bone expansion group, while the conventional drilling group showed the least bone loss at all time intervals. Pairwise comparisons between all groups were also statistically significant (p < 0.001). Intragroup analysis showed a significant increase in crestal bone loss over time within each group (p < 0.001), except for the comparison between 6 and 12 months in the conventional drilling group, which was not statistically significant (p > 0.05).

Conclusion: Implant site preparation techniques significantly influence peri-implant crestal bone remodeling. Conventional drilling demonstrated the least crestal bone loss, whereas ridge split procedures showed greater bone remodeling during the follow-up period.

## Introduction

Tooth loss is a common clinical condition resulting from dental caries, periodontal disease, trauma, and periapical pathology. Following tooth extraction, the alveolar bone undergoes progressive remodeling due to the absence of functional stimulation from the periodontal ligaments. This remodeling process often leads to a reduction in the height and width of the alveolar ridge, which may compromise the functional and esthetic rehabilitation of missing teeth [1]. Such dimensional changes in the alveolar ridge can present significant challenges during implant placement, particularly in cases in which the available bone volume is inadequate to achieve optimal implant stability.

Dental implants have become a predictable and widely accepted treatment modality for rehabilitating partially or completely edentulous patients. The long-term success of dental implants largely depends on the establishment and maintenance of osseointegration, which is defined as the direct structural and functional connection between the living bone and the surface of a load-bearing implant [2,3]. Implant success is influenced by multiple biological and mechanical factors, including bone quality, surgical technique, and patient-related variables, all of which may contribute to peri-implant bone remodeling and implant longevity. A previous study identified several predictive factors associated with dental implant failure, highlighting the importance of appropriate case selection and surgical planning to achieve favorable implant outcomes [4]. Among these, the preservation of the crestal bone around the implant neck is considered a critical parameter for maintaining implant stability, peri-implant tissue health, and long-term functional outcomes. Excessive crestal bone loss may lead to peri-implant diseases, compromised esthetics, and eventual implant failure [5].

The surgical technique used for implant site preparation plays a crucial role in determining the biological response of the bone around the implants. Conventionally, implant osteotomies are prepared using sequential drilling, a subtractive method that removes bone to create space for implant placement. While this technique provides accuracy and predictability, it may reduce the available bone volume in areas where the bone density is already compromised [6]. To overcome these limitations, alternative bone-preserving techniques, such as bone expansion (osteotome technique) and ridge split techniques, have been introduced [7,8]. The osteotome technique involves controlled lateral compression of the trabecular bone, thereby increasing bone density and improving primary implant stability, particularly in softer bone types. In contrast, the ridge split technique involves controlled splitting and expansion of the alveolar ridge to increase the ridge width and facilitate implant placement in narrow ridges [6,9].

Although these techniques are commonly employed in clinical practice, each method may influence bone remodeling and peri-implant crestal bone levels differently. Previous studies have suggested that surgical trauma, heat generation, and bone compression during osteotomy preparation can significantly affect the healing process and peri-implant bone stability [6,10]. Therefore, evaluating the influence of different implant site preparation techniques on crestal bone loss is essential for optimizing implant outcomes and improving long-term prognosis.

This study aimed to evaluate and compare crestal bone loss around dental implants placed using three different implant site preparation techniques: conventional drilling, bone expansion, and ridge split. The objectives of this study were to assess crestal bone loss around implants placed using the conventional drilling technique, evaluate crestal bone changes associated with implants placed using the bone expansion technique, and determine crestal bone loss around implants placed using the ridge split technique. Additionally, this study aimed to compare peri-implant crestal bone levels among the three surgical techniques and to evaluate peri-implant probing depth during the follow-up period to better understand the influence of implant site preparation methods on early peri-implant bone remodeling and implant stability.

## Materials and methods

The present study was designed as a prospective clinical comparative study conducted in the Department of Prosthodontics and Crown and Bridge at Shree Bankey Bihari Dental College and Research Centre, Ghaziabad, Uttar Pradesh, India, from April 2024 to September 2025. Ethical approval for the study was obtained from the Institutional Ethics Committee prior to commencement of the study (SBBDC/2024/311 dated 26-03-24). All procedures were performed in accordance with the ethical principles outlined in the Declaration of Helsinki, and written informed consent was obtained from all participants prior to their inclusion in this study.

Sample size estimation was performed using G*Power software (version 3.1, Heinrich-Heine University Düsseldorf, Germany) through an a priori analysis for one-way analysis of variance (ANOVA). Based on a previously reported effect size of 0.34 for detecting differences in crestal bone loss among implant placement techniques, with a significance level (α) of 0.05 and statistical power of 80%, the minimum required sample size was calculated to be 64 implant sites [6]. To compensate for possible dropouts or loss to follow-up, an additional 10% was added to the calculated sample size, resulting in a final sample size of 72 implant sites included in the study.

Patients requiring implant-supported rehabilitation who visited the outpatient department were screened for eligibility using clinical and radiographic examinations. A total of 72 implant sites in patients aged 25-55 years who required replacement of missing teeth with dental implants were included in this study. Patients with adequate bone height for implant placement and satisfactory oral hygiene were included in this study. Patients presenting with uncontrolled systemic conditions, severe parafunctional habits such as bruxism, active periodontal disease, heavy smoking, or inadequate bone volume requiring extensive augmentation procedures were excluded.

Preoperative evaluation included a detailed clinical examination and radiographic assessment to determine bone quality, ridge width, and anatomical considerations of the implant site. Cone-beam computed tomography (CBCT) was performed using a CBCT system (CS 8100 3D; Carestream Dental LLC, Atlanta, Georgia, United States) to evaluate bone height, ridge morphology, and proximity to adjacent anatomical structures.

Based on the implant site preparation technique used during routine clinical treatment, the implant sites were categorized into three groups, with 24 implants in each group: Group I included implant sites where osteotomy preparation was performed using the conventional sequential drilling technique (conventional drilling group). Group II consisted of implant sites where the Bone Expansion (osteotome) technique was used (bone expansion group). Group III included implant sites where the ridge split technique was performed to facilitate implant placement in narrow alveolar ridges (ridge split group). Implant placement procedures were performed as part of routine clinical treatment in the department, and the investigator recorded clinical and radiographic observations related to crestal bone levels for implants placed using these techniques. No allocation or intervention assignment was performed as part of the study design, and the study involved the evaluation of outcomes associated with techniques already used in routine clinical practice.

Commercially available titanium dental implants were used in all cases (Noris Medical Dental Implants; Noris Medical Ltd., Nesher, Haifa District, Israel). Implant site preparation was performed using a surgical kit supplied with the implant system (Noris Medical Implant Surgical Kit; Noris Medical Ltd., Nesher, Haifa District, Israel). All surgical procedures were performed under strict aseptic conditions. Local anesthesia was administered using 2% lignocaine with 1:100,000 epinephrine (Lignox 2%; Indoco Remedies Ltd., Mumbai, Maharashtra, India). A crestal incision was made in the edentulous region, followed by elevation of a full-thickness mucoperiosteal flap to expose the alveolar ridge.

In cases where adequate ridge width was present, implant osteotomy preparation was performed using the conventional sequential drilling protocol recommended by the manufacturer under copious sterile saline irrigation to minimize thermal injury to the bone. The implant was then inserted using a surgical motor unit (Implantmed Surgical Unit; W&H Dentalwerk Bürmoos GmbH, Bürmoos, Salzburg, Austria).

The bone expansion technique was employed at sites with relatively narrow ridges but adequate cancellous bone. After the initial pilot drilling, a series of bone expanders were used to gradually widen the osteotomy site and condense the surrounding bone. Expansion was performed using bone expanders from the implant system expansion kit (Bone Expander Kit; Noris Medical Ltd., Nesher, Haifa District, Israel). Following adequate expansion, the implant was placed in the prepared osteotomy.

In cases with horizontally deficient ridges where the ridge width was inadequate for implant placement using conventional drilling alone, the ridge split technique was performed. A crestal osteotomy was created along the ridge, followed by controlled splitting and gradual expansion of the cortical plates using instruments from a ridge split surgical kit (Ridge Split and Expansion Kit; Noris Medical Ltd., Nesher, Haifa District, Israel). After achieving sufficient ridge expansion, the implant osteotomy was completed, and the implant was placed.

Following implant placement, healing abutments were attached, and the surgical site was irrigated with sterile saline. The mucoperiosteal flap was repositioned and sutured with 3-0 braided silk sutures (Mersilk; Ethicon Inc., Somerville, New Jersey, United States). Postoperative instructions were provided to all patients, and standard medications, including antibiotics and analgesics, were prescribed. The sutures were removed after one week.

**Figure 1:**
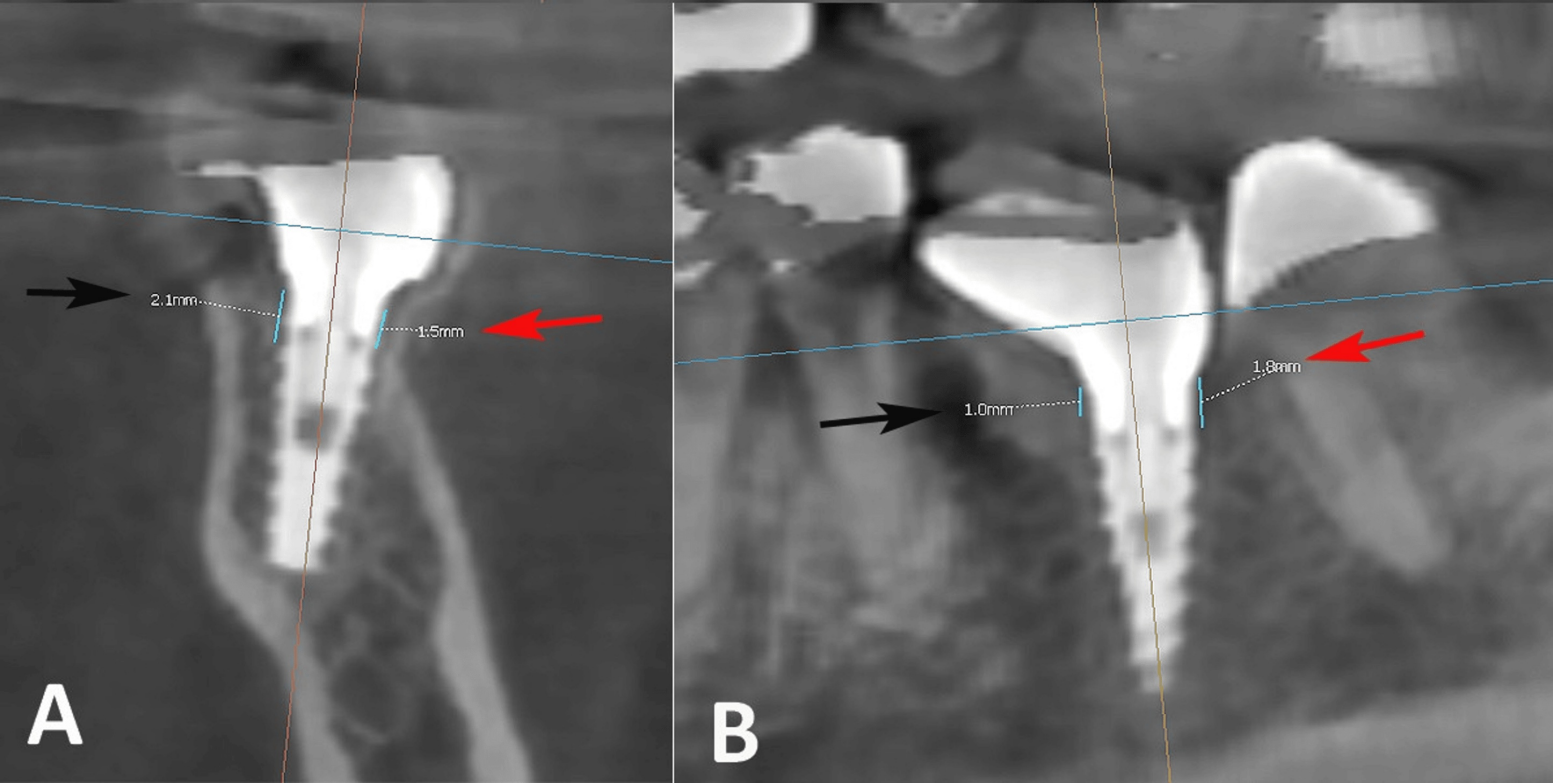
(A) CBCT coronal view of a dental implant demonstrating crestal bone loss on the buccal (red arrow) and lingual (black arrow) surfaces, (B) CBCT sagittal view showing crestal bone loss on the mesial (black arrow) and distal (red arrow) surfaces. Crestal bone loss was measured from the implant platform to the nearest apical bone–implant contact. Original CBCT images of the patient, used with the patient's permission. CBCT: Cone-beam computed tomography

Radiographic evaluation of crestal bone levels was performed using CBCT immediately after implant placement and during follow-up visits (Figure 1). Crestal bone levels were measured as the vertical distance from the implant platform to the first bone-to-implant contact on the mesial and distal aspects of the implant using measurement tools available in the CBCT software. Follow-up radiographic assessments were performed at 3, 6, and 12 months after implant placement. Measurements were recorded separately on the mesial and distal sides of the implants to evaluate changes in crestal bone levels over time.

All data were compiled and analyzed using the IBM Corp. Released 2020. IBM SPSS Statistics for Windows, Version 26. Armonk, NY: IBM Corp. Data normality was assessed and confirmed using the Shapiro-Wilk test. Descriptive statistics were expressed as means and standard deviations for continuous variables and frequencies and percentages for categorical variables. Intergroup comparisons of crestal bone loss among the three implant site preparation techniques at different follow-up intervals were performed using one-way analysis of variance (ANOVA), followed by Tukey’s post-hoc test for pairwise comparisons. Intragroup comparisons of crestal bone loss at different time intervals within each group were analyzed using repeated-measures ANOVA, followed by post-hoc Tukey analysis to identify differences between time points. Categorical variables were compared using the chi-square test. Statistical significance was set at p < 0.05.

## Results

A total of 72 implant sites were included in the study and categorized into three groups based on the implant site preparation technique: conventional drilling (CD), bone expansion (BE), and ridge split (RS), with 24 implants in each group. The demographic characteristics of the study participants are shown in Table 1. The distribution of age and sex was comparable among the three groups. Statistical analysis showed no significant differences in sex distribution or mean age among the groups, indicating that the study groups were demographically comparable at baseline.

**Table 1:** Demographic characteristics of the study groups. Values are expressed as mean ± standard deviation (SD) or frequency (percentage), *Chi-square test was used for sex distribution, whereas, **one-way analysis of variance (ANOVA) was used for age comparison.

Variable	Conventional drilling (CD) n=24	Bone expansion (BE) n=24	Ridge split (RS) n=24	Test value	p-value
Male n (%)	8 (33.5%)	8 (33.5%)	14 (58.3%)	2.06*	0.358
Female n (%)	16 (66.5%)	16 (66.5%)	10 (41.7%)		
Age, years (Mean ± SD)	38.33 ± 6.88	37.58 ± 10.46	43.08 ± 9.44	1.3**	0.285

The intergroup comparison of crestal bone loss at different follow-up intervals is shown in Table 2. Significant differences in crestal bone loss were observed among the three implant site preparation techniques at 3, 6, and 12 months for both the mesial and distal aspects. Overall, the ridge split group demonstrated the highest crestal bone loss, followed by the bone expansion group, whereas the conventional drilling group showed the least crestal bone loss across all follow-up periods.

**Table 2:** Intergroup comparison of crestal bone loss at different follow-up intervals. Values are expressed as mean ± standard deviation (mm); one-way analysis of variance (ANOVA) was used for intergroup comparisons; *Statistically significant.

Time points	Side	Conventional Drilling (CD) n=24	Bone expansion (BE) n=24	Ridge split (RS) n=24	F value	p-value
3 months	Mesial	0.22 ± 0.19	0.68 ± 0.11	1.10 ± 0.19	84.67	< 0.001*
	Distal	0.28 ± 0.19	0.78 ± 0.13	1.08 ± 0.18	66.68	< 0.001*
6 months	Mesial	0.61 ± 0.12	0.83 ± 0.17	1.50 ± 0.17	109.75	< 0.001*
	Distal	0.60 ± 0.09	0.98 ± 0.11	1.67 ± 0.16	237.03	< 0.001*
12 months	Mesial	0.69 ± 0.09	1.38 ± 0.09	2.03 ± 0.15	425.04	< 0.001*
	Distal	0.59 ± 0.13	1.22 ± 0.07	2.07 ± 0.13	501.87	< 0.001*

Pairwise comparisons between the groups using Tukey’s post-hoc test are presented in Table 3. The analysis revealed statistically significant differences between all three groups at each follow-up interval, confirming that the implant site preparation technique significantly influenced the peri-implant crestal bone levels.

**Table 3:** Post-hoc pairwise comparison of crestal bone loss among the study groups. Post-hoc Tukey’s test was used for pairwise comparisons following ANOVA, CD: Conventional drilling, BE: Bone expansion, RS: Ridge split, *Statistically significant.

Time points	Side	CD vs. BE		CD vs. RS		BE vs. RS	
		t	p	t	p	t	p
3 months	Mesial	6.87	< 0.001*	13.01	< 0.001*	6.13	< 0.001*
	Distal	7.14	< 0.001*	11.43	< 0.001*	4.29	< 0.001*
6 months	Mesial	3.59	< 0.001*	14.24	< 0.001*	10.65	< 0.001*
	Distal	7.54	< 0.001*	21.46	< 0.001*	13.92	< 0.001*
12 months	Mesial	14.94	< 0.001*	29.15	< 0.001*	14.21	< 0.001*
	Distal	13.37	< 0.001*	31.56	< 0.001*	18.19	< 0.0018

The intragroup comparison of mesial crestal bone loss over time is shown in Table 4. Repeated-measures ANOVA demonstrated a significant increase in bone loss over time within each group. Post-hoc analysis indicated that bone loss progressed significantly between the early and late follow-up intervals, with the greatest progression observed in the bone expansion and ridge split groups.

**Table 4:** Intragroup comparison of mesial crestal bone loss at different follow-up intervals. Repeated measures analysis of variance (ANOVA) was used for intragroup comparisons; post-hoc Tukey’s test was used for pairwise comparisons between time intervals; *Statistically significant.

Group	Intragroup comparison		3 months vs. 6 months		3 months vs. 12 months		6 months vs. 12 months	
	F value	p-value	t value	p-value	t value	p-value	t value	p-value
Conventional drilling (CD)	43.61	< 0.001*	7.21	< 0.001*	7.70	< 0.001*	1.82	0.288
Bone expansion (BS)	92.92	< 0.001*	3.76	0.009*	13.45	< 0.001*	8.25	< 0.001*
Ridge split (RS)	77.30	< 0.001*	4.69	0.002*	13.26	< 0.001*	7.77	< 0.001*

Similarly, the intragroup comparison of distal crestal bone loss across different follow-up intervals is presented in Table 5. A significant increase in distal crestal bone loss was observed in all groups over time. However, the progression of bone loss was more pronounced in the bone expansion and ridge split groups than in the conventional drilling group. Overall, the findings of this study indicate that the implant site preparation technique had a significant influence on peri-implant crestal bone remodeling during the follow-up period, with the conventional drilling technique demonstrating comparatively better preservation of crestal bone levels.

**Table 5:** Intragroup comparison of distal crestal bone loss at different follow-up intervals. Repeated measures analysis of variance (ANOVA) was used for intragroup comparisons; post-hoc Tukey’s test was used for pairwise comparisons between time intervals; *Statistically significant.

Group	Intragroup comparison		3 months vs. 6 months		3 months vs. 12 months		6 months vs. 12 months	
	F value	p-value	t value	p-value	t value	p-value	t value	p-value
Conventional drilling (CD)	19.62	< 0.001*	7.18	< 0.001*	4.21	0.004*	0.16	0.981
Bone expansion (BS)	54.52	< 0.001*	3.96	< 0.001*	9.64	< 0.001*	8.40	< 0.001*
Ridge split (RS)	147.47	< 0.001*	9.92	< 0.001*	18.41	< 0.001*	6.63	< 0.001*

## Discussion

Preservation of the peri-implant crestal bone is a critical determinant of long-term implant success, as excessive bone loss may compromise implant stability, soft tissue support, and esthetic outcomes. The present study evaluated and compared crestal bone loss around dental implants placed using three implant site preparation techniques: conventional drilling, bone expansion, and ridge split. The results of the present study demonstrated that implants placed using the conventional drilling technique showed the least crestal bone loss during the follow-up period, whereas the ridge split technique exhibited the greatest crestal bone loss, with the bone expansion technique showing intermediate results.

The greater crestal bone loss observed in the ridge split group can be explained by the biologic response to surgical trauma associated with this technique. Ridge splitting involves controlled cortical plate expansion and microfractures, which are known to induce the Regional Acceleratory Phenomenon (RAP), a localized, transient increase in bone turnover and remodeling. This heightened remodeling response, characterized by increased osteoclastic and osteoblastic activity, may lead to greater early crestal bone changes during the healing phase.

The relatively lower crestal bone loss observed in the conventional drilling group may be attributed to the controlled and standardized osteotomy preparation associated with this technique. Sequential drilling with copious irrigation minimizes surgical trauma and prevents thermal injury to the bone during implant site preparation. Thermal damage to the bone has been recognized as a significant factor influencing peri-implant bone remodeling, as excessive heat generation may lead to osteonecrosis and delayed osseointegration. Brisman demonstrated that an appropriate drilling speed combined with adequate irrigation plays a crucial role in reducing thermal trauma and preserving bone vitality during osteotomy preparation [11]. The minimal surgical manipulation associated with conventional drilling may contribute to improved crestal bone preservation and stable peri-implant bone levels.

The findings of the present study are consistent with the clinical observations reported by Padmanabhan and Gupta [6], who compared implants placed using conventional drilling and the osteotome technique and reported that implants placed with the conventional drilling protocol demonstrated significantly less crestal bone loss during the follow-up. Similarly, Pellicer-Chover et al. [12] reported that conventional drilling with adequate irrigation resulted in reduced peri-implant bone remodeling compared with that of alternative drilling protocols. These findings suggest that controlled osteotomy preparation remains a reliable method for preserving the peri-implant bone when an adequate ridge width is available.

The bone expansion technique demonstrated moderate crestal bone loss compared to the other groups. This finding may be explained by the biological response associated with bone condensation. The osteotome technique involves the lateral compression of the trabecular bone to increase bone density and improve primary implant stability. While this condensation of bone may enhance initial mechanical stability, it may also lead to microfractures within the trabecular bone structure, which subsequently undergoes remodeling during the healing phase. Nkenke et al. [13] reported that bone condensation with osteotomes resulted in increased bone-to-implant contact during early healing; however, this process may also initiate a greater remodeling response. Similarly, Tsolaki et al. [14] reported higher primary stability with the osteotome technique. Similarly, Shayesteh et al. [10] reported higher primary stability with osteotome techniques but observed greater marginal bone loss after one year of function than with conventional drilling. These observations support the results of the present study, in which the bone expansion group exhibited greater crestal bone loss than the conventional drilling group.

The ridge-split technique demonstrated the highest crestal bone loss among the three groups in the present study. This finding may be related to the greater surgical manipulation and cortical plate expansion involved in the ridge split procedure. Ridge splitting involves the controlled fracture and lateral displacement of cortical plates to increase the ridge width for implant placement. Although this technique allows implant placement in horizontally deficient ridges, the mechanical stress and microfractures induced during ridge expansion may lead to increased bone remodeling during the healing process. Ferrigno and Laureti [15] reported that ridge split procedures involve extensive manipulation of the cortical bone, which may result in increased crestal bone remodeling during the early healing period. Similarly, Tolstunov and Hicke [16] described that although the ridge split technique is effective for horizontal ridge augmentation, it may be associated with a higher degree of postoperative bone remodeling than conventional implant placement techniques. Jha et al. [17] systematically evaluated different devices used in ridge split procedures and highlighted that ridge expansion techniques require precise control to avoid uncontrolled fractures of the cortical plates and excessive bone trauma.

Another possible explanation for the increased bone loss observed in the ridge split group is the compromised bone quality that is often present in narrow ridges. Reduced cortical thickness and limited trabecular support may make bones more susceptible to resorption following surgical expansion. Previous literature has reported that reduced bone quantity and poor bone quality are significant risk factors associated with peri-implant bone loss and implant failure [3,4]. Therefore, implants placed in narrow ridges requiring ridge splitting may experience a more pronounced remodeling response during healing.

Despite the higher crestal bone loss observed in the ridge split group, the technique remains clinically valuable in situations where the ridge width is inadequate for conventional implant placement. Ridge splitting allows for simultaneous implant placement without the need for extensive bone grafting procedures, thereby reducing treatment duration and patient morbidity. Starch-Jensen and Becktor [18] reported that the split-crest technique provides predictable horizontal ridge augmentation with implant survival rates comparable to those achieved with bone grafting procedures. Ridge split procedures are widely used for implant placement in narrow alveolar ridges and have demonstrated predictable clinical outcomes in terms of implant stability and ridge expansion, although they may involve greater surgical manipulation of the cortical bone and subsequent remodeling [19]. Therefore, the selection of implant site preparation technique should be based on ridge morphology, bone quality, and the specific clinical scenario.

From a clinical perspective, the results of the present study highlight the importance of selecting an appropriate implant site preparation technique based on the available bone volume. Conventional drilling remains the preferred technique when adequate ridge width is present, as it provides predictable outcomes with minimal bone loss. The bone expansion technique may be beneficial for moderately narrow ridges, where preservation of the existing bone is desirable. The ridge-split technique should be reserved for cases with significant horizontal ridge deficiency, where alternative augmentation procedures may be required.

The present study had certain limitations that should be considered when interpreting the results. The sample size was relatively small, and the follow-up period was restricted to one year. Longer follow-up studies are necessary to evaluate the long-term crestal bone stability and implant survival associated with these techniques. In addition, factors such as occlusal loading, implant design, and patient-related variables may influence peri-implant bone remodeling and were not extensively evaluated in this study. Future studies with larger sample sizes, longer follow-up periods, and multicenter clinical designs are recommended to validate the findings of the present study.

## Conclusions

Within the limitations of the present study, the implant site preparation technique significantly influenced peri-implant crestal bone remodeling during the follow-up period. Implants placed using the conventional drilling technique demonstrated the least crestal bone loss and showed more favorable peri-implant bone preservation. The bone expansion technique exhibited moderate crestal bone changes, suggesting its usefulness in moderately narrow ridges while maintaining an acceptable bone stability. In contrast, the ridge split technique showed comparatively greater crestal bone loss, likely because of increased surgical manipulation and cortical plate expansion. Therefore, careful selection of the implant site preparation technique based on ridge morphology and bone quality is essential to achieve optimal implant outcomes and long-term peri-implant bone stability.
